# Epigenetic silencing of *SALL3* is an independent predictor of poor survival in head and neck cancer

**DOI:** 10.1186/s13148-017-0363-1

**Published:** 2017-06-12

**Authors:** Kiyoshi Misawa, Daiki Mochizuki, Atsushi Imai, Yuki Misawa, Shiori Endo, Masato Mima, Hideya Kawasaki, Thomas E. Carey, Takeharu Kanazawa

**Affiliations:** 1 Department of Otolaryngology/Head and Neck Surgery, Hamamatsu University School of Medicine, 1-20-1 Handayama, Hamamatsu, Shizuoka 431-3192 Japan; 2 Department of Regenerative and Infectious Pathology, Hamamatsu University School of Medicine, Hamamatsu, Japan; 3 Laboratory of Head and Neck Cancer Biology, Department of Otolaryngology/Head and Neck Surgery, University of Michigan, Ann Arbor, MI USA; 4 Department of Otolaryngology/Head and Neck Surgery, Jichi Medical University, Tochigi, Japan

**Keywords:** SALL3, Hypermethylation, Tumor-suppressor genes, Head and neck cancer, Biomarker, Real-time PCR

## Abstract

**Background:**

This study examined *Sal-like protein* (*SALL)3* methylation profiles of head and neck cancer (HNSCC) patients at diagnosis and follow-up and evaluated their prognostic significance and value as a biomarker. *SALL3* expression was examined in a panel of cell lines by quantitative reverse transcription polymerase chain reaction (RT-PCR). The methylation status of the *SALL3* promoter was examined by quantitative methylation-specific PCR.

**Results:**

*SALL3* promoter methylation was associated with transcriptional inhibition and was correlated with disease recurrence in 64.8% of cases, with an odds ratio of 1.914 (95% confidence interval: 1.157–3.164; *P* = 0.011) by multivariate Cox proportional hazard regression analysis. *SALL3* promoter hypermethylation showed highly discriminatory receiver operator characteristic curve profiles that clearly distinguished HNSCC from adjacent normal mucosal tissue, and was correlated with reduced disease-free survival (DFS) (log-rank test, *P* = 0.01). Hypermethylation of tumor-related genes was higher among patients with *SALL3* methylation than among those without methylation (*P* < 0.001). Furthermore, *SALL3* hypermethylation was associated with expression of *TET1*, *TET2*, and *DNMT3A* genes.

**Conclusions:**

This study suggests that CpG hypermethylation is a likely mechanism of *SALL3* gene inactivation, supporting the hypothesis that the *SALL3* gene may play a role in the tumorigenesis of HNSCC and may serve as an important biomarker.

**Electronic supplementary material:**

The online version of this article (doi:10.1186/s13148-017-0363-1) contains supplementary material, which is available to authorized users.

## Background

Head and neck squamous cell carcinoma (HNSCC) is a broad disease that encompasses epithelial malignancies arising in the paranasal sinuses, nasal cavity, oral cavity, pharynx, and larynx [1]. Risk factors for HNSCC development include tobacco use, alcohol consumption, sexual promiscuity, and human papilloma virus (HPV) infection [2, 3]. At least 50% of patients with locally advanced HNSCC develop local or distant failure, which is usually detected within the first 2 years of treatment [1]. To improve patient outcomes, it is necessary to identify reliable biomarkers that provide sufficient prognostic power for effective clinical management of this disease [3, 4].

*Spalt like transcription factor 3* (*SALL3*) encodes a C2H2-type zinc-finger protein in a family of evolutionarily conserved genes present in species such as *Drosophila*, *Caenorhabditis elegans*, and vertebrates [5]. Recent studies have investigated the association between *SALL3* expression and carcinogenesis. One group demonstrated that *SALL3* was silenced by DNA methylation and that the protein interacts with DNA methyltransferases 3 alpha (*DNMT3A*) in hepatocellular carcinoma [6]; another report showed that *SALL3* hypermethylation reduced the level of *SALL3* mRNA in hepatocellular carcinoma [7]; and aberrant hypermethylation of *SALL3* along with HPV infection was found to contribute to carcinogenesis in cervical cancer [8].

Loss of heterozygosity (LOH) on chromosome 18q, which is observed in a large proportion of HNSCC cases, is associated with advanced stage and decreased survival [9, 10], suggesting that one or more genes on this chromosome are important for tumorigenesis [9, 11]. The missing portion of 18q23 can vary from 53% (D18S461) to 75% (D18S70), and encompasses the *SALL3* and galanin receptor type I (*GALR1*) locus [12]. Our preliminary analyses have indicated that methylation-induced *GALR1* gene silencing is a critical event in HNSCC progression [13] associated with LOH of 18q [12], and that activation of GALR1 signaling suppresses tumor cell proliferation [14]. The findings are consistent with the notion that inactivation or loss of one or more genes on 18q contributes to aggressive tumor behavior in HNSCC.

*SALL3* promoter hypermethylation has been linked to loss of gene expression; we speculated that this a critical event in the development of HNSCC. To test this hypothesis, we investigated the methylation status of *SALL3* in 165 HNSCC cases at diagnosis and during follow-up to assess its clinical significance and potential as a prognostic biomarker for tumor recurrence and patient survival. We suggest that methylation-induced silencing of *SALL3* facilitates methylation of tumor-related genes, leading to de novo DNA methylation of *DNMT3s* and ten-eleven translocation (*TET*) family genes that potentiates enzymatic conversion of 5-methylcytosine (5mC) to 5-hydroxymethylcytosine (5hmC).

## Methods

### Tumor samples and cell lines

A total of 236 primary HNSCC specimens were obtained during surgery at the Department of Otolaryngology of Hamamatsu University School of Medicine. Clinical information including age, gender, tumor location, smoking status, alcohol consumption, tumor size, lymph node status, and tumor stage was obtained from clinical records. Tumor stage was adapted to the 7th edition of the TNM system. The mean patient age was 65.4 years (range 32–93 years), and the male: female ratio was 198:38. Primary tumors were located in the oral cavity (*n* = 73), hypopharynx (*n* = 61), larynx (*n* = 52), oropharynx (*n* = 41), and nasal cavity (*n* = 9). The primary endpoints of this study were disease-free survival (DFS). A total of 165 primary HNSCC patients were surveilled for up to 3 years after initial treatment. Patients provided written, informed consent for participation in the study and the protocol was approved by the Institutional Review Boards at the Hamamatsu University School of Medicine. DNA and cDNA from 11 University of Michigan squamous cell carcinoma (UM-SCC) cell lines, 99 F fibroblasts, and HOK-16B cells were provided by Dr. Thomas E. Carey of the University of Michigan. Normal human keratinocytes (NHK) were a gift from Dr. No Hee Park of the University of California at Los Angeles School of Dentistry [15]. For reactivation of *SALL3* expression, cultures were incubated for 48 h with 5-azacytidine (15 μg/ml, A2385; Sigma-Aldrich, St. Louis, MO, USA), a DNA methyltransferase inhibitor [13].

### Bisulfite modification and quantitative methylation-specific PCR (qMSP)

**Fig. 1:**
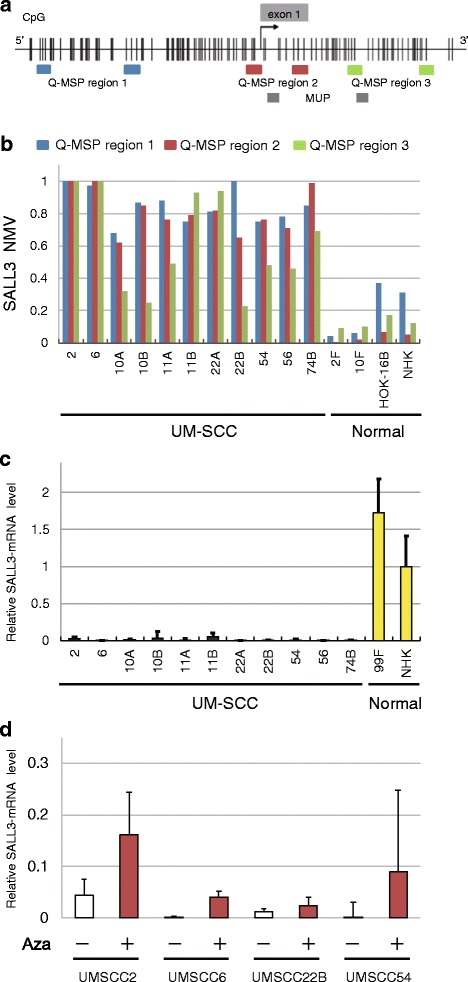
Schematic representation of SALL3 gene methylation analysis by qMSP and evaluation of SALL3 expression by qRT-PCR in UM-SCC cell lines. **a** *Colored boxes* indicate the three regions examined by qMSP (P1, *blue box*; P2, *red box*; P3, *green box*). Grayed boxes indicate the regions examined by MUP. The *bent arrow* indicates the TSS. CpG sites are indicated by black vertical lines. The *gray box* denotes the exon 1. **b** qMSP of the *SALL3* promoter region in HNSCC cell lines. Genomic DNA of non-malignant cells was included as a control (P1, *blue box*; P2, *red box*; P3, *green box*). **c** Relative mRNA expression level of *SALL3*, as determined by qRT-PCR in 11 UM-SCC and two normal cell lines. **d** Effect of 5-azacytidine on *SALL3* expression in four cell lines with densely methylated *SALL3*, as evaluated by qRT-PCR. Controls were cells were similarly treated but without 5-azacytidine

Bisulfite modification of genomic DNA was carried out as previously described. [16] *SALL3* promoter methylation was assessed by qMSP with the TP800 Thermal Cycler Dice Real-Time System (Takara Bio, Otsu, Japan) using the primer sequences shown in Additional file 1: Table S1. *SALL3* CpG islands and regions analyzed by qMSP are shown in Fig. 1a. We tested three different primer sets to identify the pair that would most reliably predict DNA methylation. *SALL3* region 1, 2, and 3 primers amplified sequences upstream of, around, and downstream of the transcription start site (TSS), respectively (i.e., P1, P2, and P3, respectively). A standard curve was generated using serial dilutions of EpiScope Methylated HeLa genomic DNA (3520; Takara), with fully methylated (FM) DNA used as a control. The normalized methylation value (NMV) was defined as follows: NMV = (*SALL3*-S/*SALL3*-FM)/(*ACTB*-S/*ACTB*-FM), where *SALL3*-S and *SALL3*-FM represent *SALL3* methylation levels in sample and universal methylated DNAs, respectively, and *ACTB*-S and *ACTB*-FM correspond to β-actin in sample and universal methylated DNAs, respectively [17]. For amplification reactions, 2 μL (0.01 μg/μL) of bisulfite treatment of genomic DNA, 12.5 μL of SYBR® Premix DimerEraser TM Perfect Real Time (TaKaRa, Tokyo, Japan), and 0.5 μL (10 μM) of each primer were added to a final volume of 25 μL. The PCR conditions were as follows: one denaturing cycle at 95 °C for 10 s, followed by 40 cycles of denaturing at 95 °C for 5 s, and annealing/extension at 58 °C for 30 s (two-step reaction). Dissociation curves are carried out at the end of a PCR experiment by following a 3-step procedure (Additional file 2: Figure S1).

### MSP/unmethlylation-specific PCR (UMSP) analysis and methylation-unspecific qPCR (MUP) assay

Bisulfite-treated DNA was PCR-amplified using two MSP/UMSP primer and MUP primer pairs targeting the *SALL3* gene promoter region; the sequences are provided in Additional file 1: Table S1. The PCR conditions were 94 °C for 5 min; 39 cycles at 94 °C for 30 s, 58 °C (MSP primer for methylated DNA detection) or 54 °C (UMSP primer for unmethylated DNA detection) for 30 s, and 72 °C for 40 s; and 72 °C for 5 min. The 106-bp PCR products were separated by gel electrophoresis on a 9% polyacrylamide gel and visualized by staining with ethidium bromide.

### Methylation analysis by droplet digital (dd) PCR

*SALL3* methylation was confirmed using qMSP P2 primers and the QX200 Droplet Digital PCR system (1864001JA; Bio-Rad, Hercules, CA, USA); the reaction was run in duplicate and samples were transferred one column at a time to an 8-channel Droplet Generator Cartridge (1864008; Bio-Rad). Droplet Generator Oil (1863005; Bio-Rad) was then added, and droplets were produced using a QX200 Droplet Generator (1864002; Bio-Rad). After amplification, the PCR plate was placed in the QX200 Droplet Reader (1864003; Bio-Rad) and droplets were subsequently confirmed as being either positive or negative for fluorescence/amplification. Data were analyzed using QuantaSoft software (18640011; BioRad).

### RNA extraction and quantitative reverse transcription (qRT-)PCR of SALL3, DNMT3A, DNMT3B, TET1, TET2, and TET3

Total RNA was isolated with RNeasy Plus Mini kit (74134; Qiagen, Valencia, CA, USA), and cDNA was synthesized with ReverTra Ace qPCR RT kit (FSQ-101; Toyobo, Osaka, Japan). [17] Primer sequences are shown in Additional file 1: Table S1. Target mRNA expression was compared between samples by normalization to *glyceraldehyde 3-phosphate dehydrogenase* (*GAPDH*) mRNA expression.

### The Cancer Genome Atlas (TCGA) data

DNA methylation and mRNA expression data for invasive HNSCC were collected in July 2016 from MethHC, a database of DNA methylation and gene expression in human cancer (http://methhc.mbc.nctu.edu.tw/php/index.php) [18]. DNA methylation data obtained using the Infinium HumanMethylation450 platform (Illumina, San Diego, CA, USA) are shown as β values.

### Analysis of high-risk HPV status

The HPV status was evaluated using the HPV Typing Set (Takara Bio., Tokyo, Japan), a PCR primer set specifically designed to identify HPV genotypes −16, −18, −31, −33, −35, −52, and −58 in genomic DNA. The PCR HPV Typing Set method was performed according to the manufacturer’s protocol. The PCR products were separated using 9% polyacrylamide gel electrophoresis and stained with ethidium bromide.

### Data analysis and statistics

Receiver operating characteristic (ROC) curve analysis was performed using NMVs for the 36 HNSCC specimens and adjacent normal mucosal tissue samples. Differences in *SALL3* methylation levels between tumor and normal tissues were compared by the paired t test. A Spearman correlation analysis was performed to evaluate interactions between *SALL3* expression and methylation of each primer set. The association between discrete variables and *SALL3* methylation was tested by Fisher’s exact probability and Student *t* tests. DFS curves were constructed by the Kaplan-Meier method and were evaluated by the log-rank test. Cox’s proportional hazards regression analysis for age, gender, smoking status, alcohol consumption, tumor stage, and *SALL3* methylation status was performed to determine the multivariate predictive value of prognostic factors. Differences were considered as significant when the probability was less than 0.05. Statistical analyses were performed using Stat-Mate IV (ATMS Co., Tokyo, Japan).

## Results

### *SALL3* expression in UM-SCC cells is correlated with promoter methylation

To determine whether the *SALL3* promoter is methylated in HNSCC, qMSP analysis was carried out on three regions of bisulfite-treated DNA from UM-SCC cell lines, fibroblasts, and NHKs. In all cancer cell lines with reduced *SALL3* expression, the NMV of *SALL3* was higher than that in normal cell lines (Fig. 1b); moreover, the absence of *SALL3* expression was associated with hypermethylation. In contrast, normal fibroblasts and NHKs were *SALL3*-positive (Fig. 1c). Treatment with 5-azacytidine resulted in upregulation of *SALL3* (Fig. 1d).

**Fig. 2:**
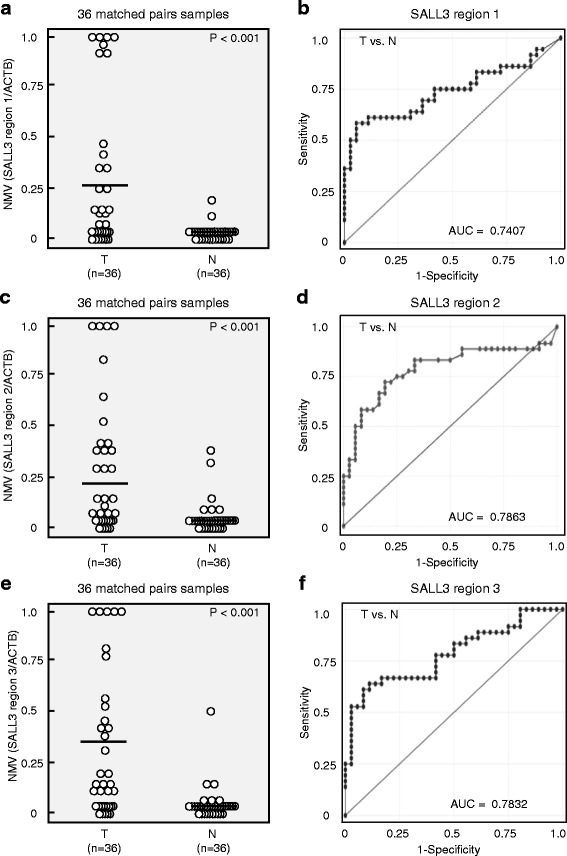
Pattern of methylation in matched pairs of head and neck tumors and adjacent normal mucosal tissues, and ROC curve analysis of NMV. **a** SALL3-P1 NMVs of head and neck tumors were higher than those of adjacent normal mucosal tissues (*P* < 0.001). **b** Area under the ROC curve (AUROC) for *SALL3*-P1 was 0.7407. **c** A higher frequency and degree of *SALL3*-P2 methylation was observed in head and neck tumors than in matched normal mucosae (*P* < 0.001). **d** AUROC value for *SALL3*-P2 was 0.7863. At the cutoff value of 0.11, sensitivity was 58.3% and specificity was 91.7%. **e** *SALL3*-P3 NMVs of head and neck tumors were higher than those of paired adjacent normal mucosae (*P* < 0.001) **f** AUROC value for *SALL3*-P3 was 0.7832

*SALL3* promoter methylation status was analyzed in 36 cancerous and paired noncancerous mucosae by qMSP. Promoter methylation levels were represented by NMVs, which is the ratio of methylated DNA at the target sequence in each specimen to fully methylated control DNA. *SALL3* methylation status was assessed using three sets of primers (P1: median NMV = 0.269 vs. 0.030, *P* < 0.001; P2: median NMV = 0.285 vs. 0.056, *P* < 0.001; P3: median NMV = 0.314 vs. 0.058, *P* < 0.001) (Fig. 2a, c, e). The ROC curves showed similar areas under the curve (AUC) (P1: 0.7407, P2: 0.7863, and P3: 0.7832) (Fig. 2b, d, f). The Spearman analysis revealed that *SALL3* expression in 83 HNSCC cases and paired normal tissue was negatively correlated with P1 NMV (*ρ* = −0.1882, *P* = 0.0160), P2 NMV (*ρ* = −0.2835, *P* = 0.0019), and P3 NMV (*ρ* = −0.192, *P* = 0.0211) (Additional file 3: Figure S2A–C). P2 showed the maximum AUC and Spearman’s rho. The cutoff NMV (0.11) for P2 was identified from the ROC curve to maximize sensitivity (58.3%) and specificity (91.7%) (Fig. 2d).

### *SALL3* methylation status in tumor samples

Among 236 DNA samples from untreated primary tumors examined with P2, a specimen was classified as methylated when the NMV exceeded 0.11. The *SALL3* promoter was methylated in 153/236 (64.8%) cases and unmethylated in 83/236 (35.2%) cases. There was no association between *SALL3* promoter hypermethylation and other clinical characteristics (Table 1, Additional file 4: Figure S3).

**Table 1:** *SALL3* gene methylation status in primary samples of HNSCC

Patient and tumor characteristics (*n* = 236)	Methylation		
	Present (*n* = 153)	Absent (*n* = 83)	*P* value
Age^a^			
70 and older (82)	56	26	0.475
Under 70 (154)	97	57	
Sex^a^			
Male (198)	126	72	0.460
Female (38)	27	11	
Smoking status^a^			
Smoker (174)	116	58	1
Nonsmoker (62)	37	25	
Alcohol intake^a^			
Ever (172)	112	60	1
Never (64)	41	23	
Tumor size^a^			
T1-2 (109)	71	38	1
T3-4 (127)	82	45	
Lympho-node status^a^			
N0 (102)	67	35	0.891
N+ (134)	86	48	
Stage^a^			
I, II, III (105)	68	37	1
IV (131)	85	46	
HPV status			
Positive (29)	18	11	1
Negative (199)	130	69	
Indeterminate (8)			

^a^Fisher’s exact probability test

### *SALL3* promoter methylation predicts poor patient outcome

**Fig. 3:**
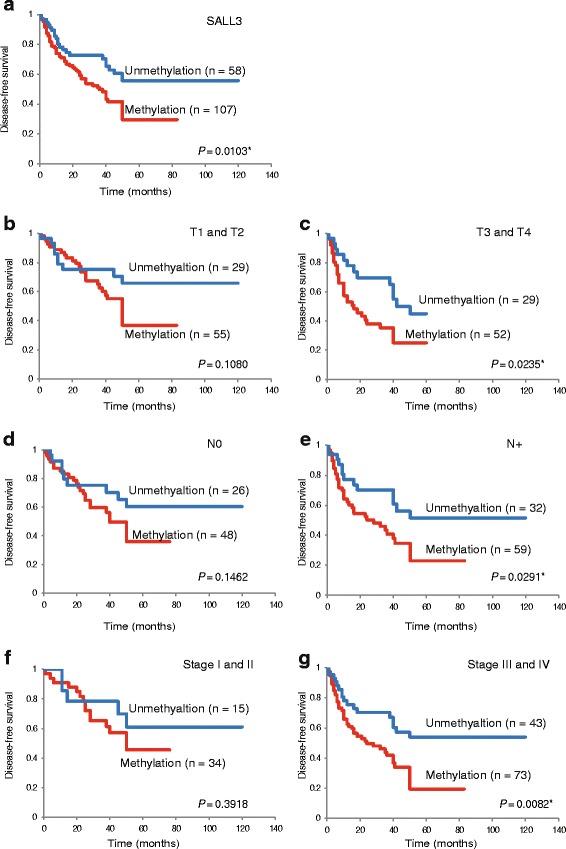
Kaplan-Meier survival curves for HNSCC patients. DFS for (**a**) all 165 HNSCC cases, (**b**) tumor size in T1 and T2 cases (*n* = 84), (**c**) tumor size T3 and T4 cases (*n* = 81), (**d**) lymph node status N0 cases (*n* = 74), (**e**) lymph node status N+ cases (*n* = 91), (**f**) stage I and II cases (*n* = 49), and (**g**) stage III and IV cases (*n* = 116). *Gray and black lines* indicate patients without and with methylation, respectively

Kaplan–Meier plots revealed that methylation of *SALL3* and other genes in 165 tumors were related to DFS duration in some patients (log-rank test, *P* = 0.01) (Fig. 3a). *SALL3* methylation status in patients with T1 and T2, without lymph node metastasis, or Stage I and II tumors was not related to outcome (Fig. 3b, d, f). Patients with T3 and T4, lymph node metastasis, and Stage III and IV tumors had decreased DFS as compared to those without *SALL3* methylation (*P* = 0.024, *P* = 0.029, and *P* = 0.008, respectively) (Fig. 3c, e, g). Kaplan-Meier survival curves using median methylation as a cut-off was not statistically significant (Additional file 5: Figure S4A, B). In other words, patients with advanced cancer had worse prognosis. In addition, results of the multivariate Cox proportional hazard regression analysis—which included age, gender, alcohol consumption, smoking status, and tumor stage—indicated that survival rates were 1.914 times lower in patients with *SALL3* gene methylation than in those without methylation (*P* = 0.011) (Table 2). Furthermore, Cox’s proportional hazards regression analysis including HPV status was lower in patients with *SALL3* gene methylation than in those without methylation (*P* = 0.009) (Additional file 6: Table S2). These results indicate that *SALL3* promoter methylation is a predictor of poor outcome in HNSCC patients.

**Table 2:** Multivariate analysis of factors affecting survival using Cox proportional hazards model in 165 HNSCC patients

	Disease-free survival	
Variables	HR (95% CI)	*P*
Age		
70 and older vs. <70	1.062 (0.659–1.711)	0.8038
Gender		
Male vs. female	1.170 (0.644–2.125)	0.6064
Alcohol exposure		
Ever vs. never	1.015 (0.565–1.823)	0.9606
Smoking status		
Smoker vs. nonsmoker	1.339 (0.752–2.383)	0.3216
Stage		
I, II, III, vs. IV	2.356 (1.480–3.751)	0.0003*
*SALL3* methylation		
Yes vs. no	1.914 (1.157–3.164)	0.0114*

*HR* hazard ratio, *95% CI* 95% confidence interval

### *SALL3* promoter methylation is associated with HNSCC by MSP/UMSP, ddMSP analyses and methylation-unspecific qPCR (MUP) assay

Primary tumor samples were tested with both sets of primers (MSP and UMSP) for P2. All 12 samples that showed promoter hypermethylation by qMSP (Samples 5, 6, 14, 37, 39, 40, 43. 46, 55, 59, 61, and 64) had predominantly methylated alleles (Additional file 7: Figure S5A). Cancerous and paired noncancerous mucosae were also analyzed by the ddMSP assay; the results showed that all cancerous samples (T) in which *SALL3* methylation was detected by qMSP showed positive signals. On the other hand, most noncancerous mucosa samples (N) showed no *SALL3* methylation (Additional file 7: Figure S5B). A MUP assay targeting a CpG-free gene locus close to *SALL3* as a reference assay showed positive signals (Additional file 7: Figure S5C).

### Methylated SALL3 along with other molecular markers is associated with patient survival

We evaluated the methylation status of deleted in colorectal cancer (*DCC*), galanin receptor (*GALR*)1, *p16*, Ras association domain family 1 isoform A, E-cadherin (*CDH1*), H-cadherin (*CDH13*), O(6)-methylguanine-DNA methyltransferase (*MGMT*), and death-associated protein kinase (*DAPK*) genes, which are involved in methylation. *SALL3* methylation was correlated with *GALR1*, *CDH1*, *CDH13*, and *DAPK* methylation (*P* < 0.001, *P* = 0.002, *P* = 0.005, and *P* < 0.001, respectively) (Additional file 8: Table S3). Hypermethylation of tumor-related genes was higher among patients with *SALL3* methylation than among those without methylation (*P* < 0.001) (Additional file 9: Figure S6).

### *SALL3* methylation level and TET and DNMT3 expression in HNSCC specimens

**Fig. 4:**
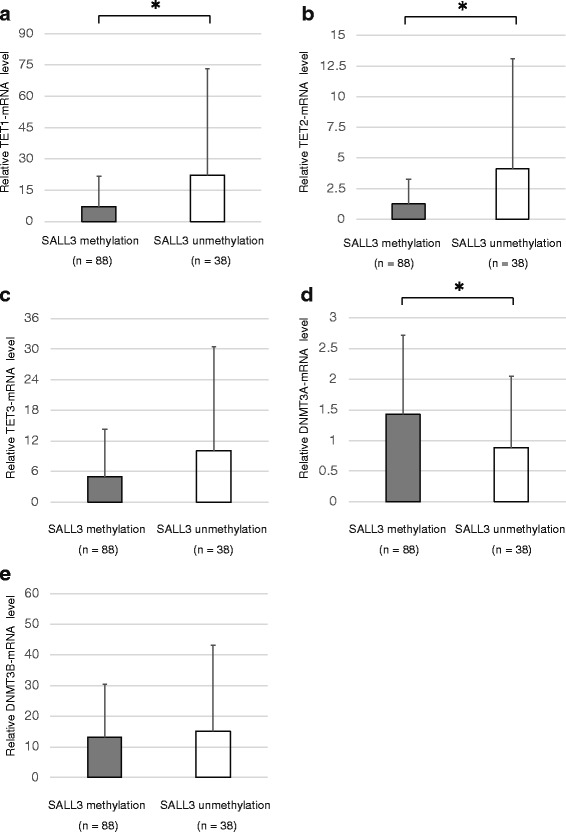
Correlation between SALL3 methylation status and TET and DNMT3 mRNA expression in HNSCC patients. **a** *TET1* expression. **b** *TET2* expression. **c** *TET3* expression. **d** *DNMT3A* expression. **e** *DNMT3B* expression

We examined *TETI*, *TET2*, *TET3*, *DNMT3A*, and *DNMT3B* mRNA levels by qRT-PCR. *TET1*, *TET2*, and *DNMT3A* expression was associated with *SALL3* methylation levels (*P* = 0.011, 0.015, and 0.027, respectively) (Fig. 4a, b, d). However, *TET3* and *DNMT3B* expression was not associated with *SALL3* methylation level (*P* = 0.064 and *P* = 0.645, respectively) (Fig. 4c, e).

### External validation of TCGA data

HNSCC data from TCGA were examined for *SALL3* DNA methylation. *SALL3* methylation showed an average β value of 0.350 in the HNSCC TCGA cohort as compared to 0.098 in normal samples (*P* < 0.001, Student’s t test) (Additional file 10: Figure S7A). Consistent with our data, there was a strong negative correlation between *SALL3* DNA methylation and expression (Additional file 10: Figure S7B). Together with our previous finding that the region around the TSS of the *SALL3* gene exhibits promoter activity, these results suggest that HNSCC is characterized by epigenetic silencing of *SALL3* via promoter hypermethylation. Furthermore, *SALL3* DNA methylation was significantly associated with age and smoking status (*P* = 0.007 and *P* = 0.030, respectively) (Additional file 11: Table S4).

Moreover, using mRNA expression data, *p16*-negative tumors were significantly lower *SALL3* mRNA expression than *p16*-positive tumors (5.23 ± 15.94 vs. 63.39 ± 117.38, *P* < 0.001) (Additional file 12: Table S5). Kaplan–Meier analysis revealed that the *SALL3* methylation and expression levels were not significantly associated with the overall survival. (Additional file 13: Figure S8) Furthermore, the methylation status of the other eight individual genes was not associated with outcome (Additional file 14: Figure S9).

## Discussion

Clarifying the epigenetic regulation of *SALL3* can provide insights into the mechanisms of tumorigenesis and the risk of disease recurrence. To this end, the present study investigated *SALL3* promoter methylation profiles in 236 HNSCC patient tissues. We found that hypermethylation of CpG islands in the *SALL3* promoter was independently associated with aggressive tumor behavior, suggesting that *SALL3* acts as a tumor suppressor gene and can serve as a prognostic biomarker in HNSCC.

*SALL3* encodes a spalt-like homeoprotein containing double zinc-finger domains. The *SALL3* locus on human chromosome 18q23 is likely included in 18q deletion syndrome [19]. In mouse, *Sall3* heterozygous knockouts (*Sall3* ^+/−^) are indistinguishable from wild-type littermates and are fertile, while homozygous mutants (*Sall3* ^−/−^) fail to survive after 12 h of extrauterine life [20]. Absence of *Sall3* may lead to palate deficiency, cranial nerve abnormalities, and perinatal lethality [20]. In humans, this deletion is associated with hearing loss, cardiac defects, mental retardation, midfacial hypoplasia, delayed growth, and limb abnormalities [21, 22].

Three additional *SALL* genes have been identified thus far: *SALL1* on chromosome 16q12.1, *SALL2* on chromosome 14q11.2, and *SALL4* on chromosome 20q13.2 [20]. *SALL1* hypermethylation has been described in several malignancies, including non-small cell lung cancer, prostate tumors [23], chronic lymphocytic leukemia [24], and acute lymphoblastic leukemia [25]. SALL2 binds to the neurotrophin receptor and regulates neuronal development [26]. LOH in the region of 14q12 harboring the *SALL2* gene and *SALL2* promoter methylation have been reported in ovarian cancer [27]. *SALL4* is important for maintaining a pluripotent state in mouse embryonic stem cells [28–30], and hypomethylation of the promoter is a common event in myelodysplastic syndrome [31]. The presence of epigenetic repressors and/or the enzymatic activities of DNMT, methyl-CpG-binding domain protein 2, and histone deacetylase 1 are associated with *SALL4* repression [32].

Exposure to carcinogens such as HPV, tobacco, and alcohol is associated with epigenetic gene inactivation in human cancers, including head and neck cancer [33]. Recently, oncogenic viruses such as HPV and Epstein-Barr and hepatitis viruses were found to induce oncogenic changes to the DNA methylome by increasing the expression and activity of DNMT3A [34–36]. DNMTs play an important role in genomic integrity, disruption of which may result in chromosomal instability and tumor progression [37]. DNMT levels—especially those of DNMT3A and DNMT3B—are often increased in various cancer tissues and cell lines, which may partly account for the hypermethylation of CpG-rich regions in tumor suppressor gene promoters in various malignancies [38]. More recently, methyl donor depletion significantly increased expression of *DNMT3A*, *TET*, and key pro-apoptotic genes such as *DAPK* [39].

TET proteins regulate the dynamic conversion of cytosine (C), 5mC, and 5hmC, and consequently, the balance between DNA methylation and demethylation [40]. Missense and truncating mutations in *TET* genes are present in nearly all solid tumor types at a relatively low frequency [41]. It was recently reported that *TET2* expression is lower in esophageal SCC than in normal epithelium and associated with 5-hmC expression; it was speculated that *TET2* is more significant in esophageal SCC development than *TET1* and *TET3* [42]. There is little known about the role of *TET* family genes in HNSCC.

The DNA methylation profile of a cell is maintained by both the DNA methylation and demethylation pathways [43]. DNA methylation and demethylation occurs either passively or actively. Alterations in 5mC writers, readers, and modifiers that affect their level, all potential mechanisms contributing to altered chromatin composition and structure as well as genome activity and stability and contribute to an overwhelming variety of human diseases [44].

*SALL3* is silenced by DNA methylation and the protein directly interacts with DNMT3A in human hepatocellular carcinoma; *SALL3* inactivation by DNA methylation was found to accelerate aberrant DNA methylation [6]. Furthermore, suppressed expression of histone methyltransferases, which in turn resulted in a decrease of di- and trimethylated H3K9 around *SALL3* genes’ promoter [45]. HPV infection was positively associated with hypermethylation of the *SALL3* promoter, and *SALL3* mRNA level was lower in HPV-positive as compared to -negative cervical cancer tissues [8]. *SALL3* hypermethylation has been reported in several cancers, including in cervical [8] and breast [46] cancers and hepatocellular carcinoma [7]. Thus, detecting aberrant *SALL3* methylation can serve as a means of identifying patients at high risk of relapse.

This study suggests that CpG hypermethylation is a likely mechanism of *SALL3* gene inactivation, supporting the hypothesis that the *SALL3* gene may play a role in the tumorigenesis of HNSCC and may serve as an important biomarker. We demonstrated for the first time that *SALL3* mRNA is downregulated in HNSCC owing to DNA methylation; this may be a critical event in HNSCC progression that is associated with DFS. Indeed, *SALL3* promoter methylation was increased in tumor tissue as compared to that in noncancerous mucosae from the same patient. *SALL3* promoter methylation was associated with disease recurrence. Moreover, transcriptional inactivation of *SALL3* was associated with aberrant methylation of other tumor-related genes and *TET1*, *TET2*, and *DNMT3A* levels in HNSCC. Our findings suggest that these methylation markers can be used in clinical practice to identify patients that may benefit from adjuvant therapy after initial surgical treatment; however, this must be confirmed in additional prospective studies in other HNSCC patient groups.

## Conclusion

The present study showed that the SALL3 promoter methylation profile appears to be an important marker predicting the clinical outcome of HNSCC. This demonstrates that molecular stratification may predict cancer progression. These findings can benefit HNSCC screening and surveillance algorithms.

## Additional files

Additional file 1: Table S1. Q-MSP primer, MSP/UMSP primers, MUP primer, and Q-RT primer list (DOCX 30 kb).

Additional file 2: Figure S1. Standard curve plot showing Ct versus initial quantity and dissociation curves*.* (A) Efficiency of primers of *SALL3* gene checked for methylation quantification using dilutions of universal methylated DNAs control. (B) Dissociation curves are carried out at the end of a PCR experiment by following a 3-step procedure (EPS 1413 kb).

Additional file 3: Figure S2. Spearman rank correlation between SALL3 expression and promoter methylation status using three SALL3 primer sets*.*(A) *SALL3* mRNA expression was inversely correlated with *SALL3-*P1 hypermethylation (correlation coefficient = 0.1882, *P* = 0.0160). (B) *SALL3-*P2, correlation coefficient = 0.2835, *P* = 0.0019. (C) *SALL3-*P3, correlation coefficient = 0.192, *P* = 0.0211. The Spearman rank correlation for *SALL3* obtained using the *SALL3-*P2 primer pair was used to maximize the correlation coefficient (EPS 1299 kb).

Additional file 4: Figure S3. Comparison of normalized methylation value (NMV) amongst selected clinical parameters. The mean NMVs for the different groups were compared by using Student’s *t*-test. *P < 0.05 (EPS 952 kb).

Additional file 5: Figure S4. Kaplan-Meier survival curves for HNSCC patients using median methylation as a cut-off. (A) DFS for all 165 HNSCC cases, high methylation group versus low methylation group. (B) Combined analyses of *SALL3* methylation status and HPV status; Hme, high methylation; Lme, low methylation; HPV (+), HPV positive; HPV (−), HPV negative (EPS 797 kb).

Additional file 6: Table S2. Multivariate analysis of factors affecting survival using Cox proportional hazards model in 157 HNSCC patients (DOCX 15 kb).

Additional file 7: Figure S5. MSP/UMSP, ddPCR assays and MUP assay. (A) Representative results from the MSP/UMSP assay of *SALL3* expression in primary HNSCC showing methylated samples (no. 5, 6, 14, 37, 39, 40, 43, 46, 55, 59, 61, and 64). (B) Representative ddMSP results for tumor (T) and normal (N) samples. (C) Representative MUP results for tumor (T) samples (EPS 1790 kb).

Additional file 8: Table S3. *SALL3* Gene Methylation Status in Primary Samples of HNSCC with the methylation of other eight genes (DOCX 21 kb).

Additional file 9: Figure S6. Correlation with other tumor-related genes. Comparison of methylation rates in eight genes along with *SALL3* in primary HNSCC (EPS 609 kb).

Additional file 10: Figure S7.SALL3 DNA methylation and expression data for HNSCC from TCGA database. (A) *SALL3* DNA methylation profiles of HNSCC and normal tissue samples (*P* < 0.001). (B) Spearman rank correlation coefficient ($\rho$) and *P* values are shown. An inverse correlation was observed between *SALL3* methylation and expression in HNSCC (EPS 1321 kb).

Additional file 11: Table S4. *SALL3* Gene Methylation levels in TCGA cohort of HNSCC (DOCX 21 kb).

Additional file 12: Table S5. *SALL3* mRNA levels in TCGA cohort of HNSCC (DOCX 23 kb).

Additional file 13: Figure S8. Overall survival based on SALL3 gene signatures in the TCGA cohort using median methylation as a cut-off. (A) *SALL3* DNA methylation profiles of HNSCC. (B) *SALL3* mRNA expression profiles of HNSCC. Patients were divided into 2 groups. (EPS 884 kb)

Additional file 14: Figure S9. Overall survival curves of other tumor-related genes in the TCGA cohort using median methylation as a cut-off. Overall survival curves of (A) *DCC*, (B) *GALR1*, (C) *p16*, (D) *RASSF1A*, (E) *E-cadherin*, (F) *H-cadherin*, (G) *MGMT* and (H) *DAPK*. Patients were divided into two groups (EPS 1548 kb).

## Abbreviations

ACTB: β-actin
ddPCR: Droplet digital PCR
DFS: Disease-free survival
DNMT3: DNA methyltransferases 3
GALR1: Galanin receptor type I
GAPDH: Glyceraldehyde 3-phosphate dehydrogenase
HNSCC: Head and neck squamous cell cancer
HPV: Human papilloma virus
LOH: Loss of heterozygosity
NHK: Normal human keratinocytes
NMV: Normalized methylation value
qMSP: Quantitative methylation-specific PCR
ROC: Receiver operating characteristic
SALL3: Sal-like protein 3
TCGA: The Cancer Genome Atlas
TET: Ten-eleven translocation
TSS: Transcription start site
UMSP: Unmethlylation-specific PCR
