# Supplementary material for: Epigenetic silencing of SALL3 is an independent predictor of poor survival in head and neck cancer
Source: Clin Epigenetics. 2017 Jun 12;9:64. doi: 10.1186/s13148-017-0363-1 (PMC5469057; doi:10.1186/s13148-017-0363-1)
Supplement: Supplementary file 1 — Q-MSP primer, MSP/UMSP primers, MUP primer, and Q-RT primer list (DOCX 30 kb). [file 13148_2017_363_MOESM1_ESM.docx]

**Additional file 1: Table S1. Q-MSP primer, MSP/UMSP primers, MUP primer and Q-RT primer list**

PCR

Q-MSP

Q-MSP (MSP)

UMSP

Q-MSP

Q-MSP

MUP

Q-RT

Q-RT

Q-RT

Q-RT

Gene

SALL3 Position 1

SALL3 Position 2

SALL3 Position 2

SALL3 Position 3

ACTB

SALL3

SALL3

DNMT3A

DNMT3B

TET1

Forward/Reverse

Forward

Reverse

Forward

Reverse

Forward

Reverse

Forward

Reverse

Forward

Reverse

Forward

Reverse

Forward

Reverse

Forward

Reverse

Forward

Reverse

Forward

Reverse

Base pairs

135

106

106

122

133

192

180

108

154

81

Sequence (5’-3’)

ATGTGAGGCGGCGTCGGGTA

CCGATTAACCGAACTCCAACG

GGGGTTCGAGCGTCGTTAGT

CCGTACTCGAAAACCCCGTC

GGGGTTTGAGTGTTGTTAGT

CCATACTCAAAAACCCCATC

CGTCCGGGAGCGGGAGAAAG

CGCCCACAACTCTCTCGACG

TGGTGATGGAGGAGGTTTAGAAGT

AACCAATAAAACCTACTCCTCCCTTAA

GTAAGTAGGTTAAGTTTTAGT

CAAAAAACAAAAAACTTTCTCCC

ACGCCCAAGCAGCACAACT

GGCGTTATTCCACATGTGTGTC

AGTACGACGACGACGGCTA

CACACTCCACGCAAAAGCAC

AGGGAAGACTCGATCCTCGTC

GTGTGTAGCTTAGCAGACTGG

CCCTTGGAAATGCCATAGGAA

GAGAGCCTGCTGGAACTGTTTG

PCR

Q-RT

Q-RT

Q-RT

Gene

TET2

TET3

GAPDH

Forward/Reverse

Forward

Reverse

Forward

Reverse

Forward

Reverse

Base pairs

117

81

138

Sequence (5’-3’)

GGCTGTTGGCCAGAGACTTA

ATACCTGTAGGTGTTTGCCTGTTTA

GCCAACTTCAACATACCCTGGAC

CACCTGGATGTGGGACTGTGTAA

GCACCGTCAAGGCTGAGAAC

TGGTGAAGACGCCAGTGGA
