# Supplementary material for: Epigenetic silencing of SALL3 is an independent predictor of poor survival in head and neck cancer
Source: Clin Epigenetics. 2017 Jun 12;9:64. doi: 10.1186/s13148-017-0363-1 (PMC5469057; doi:10.1186/s13148-017-0363-1)
Supplement: Supplementary file 6 — Multivariate analysis of factors affecting survival using Cox proportional hazards model in 157 HNSCC patients (DOCX 15 kb). [file 13148_2017_363_MOESM6_ESM.docx]

**Table S2. Multivariate analysis of factors affecting survival using Cox proportional hazards model in 157 HNSCC patients.**

Disease-free survival

Variables HR (95% CI) *P*

## Age

70 and older vs. <70

## HPV

Positive vs. Negative

## Alcohol exposure

Ever vs. Never

## Smoking status

Smoker ve. Non smoker

## Stage

I, II, III vs. IV

## SALL3 methylation

Yes vs. No

HR: hazard ratio

95% CI: 95% confidence interval

1.093 (0.664-1.798)

0.501 (0.216-1.159)

0.964 (0.532-1.747)

1.388 (0.749-2.570)

2.395 (1.465-3.916)

2.008 (1.187-3.396)

0.7264

0.1062

0.9045

0.2972

0.0005*

0.0094*
