# Supplementary material for: Epigenetic silencing of SALL3 is an independent predictor of poor survival in head and neck cancer
Source: Clin Epigenetics. 2017 Jun 12;9:64. doi: 10.1186/s13148-017-0363-1 (PMC5469057; doi:10.1186/s13148-017-0363-1)
Supplement: Supplementary file 8 — SALL3 Gene Methylation Status in Primary Samples of HNSCC with the methylation of other eight genes (DOCX 21 kb). [file 13148_2017_363_MOESM8_ESM.docx]

**Table S3.** ***SALL3* Gene Methylation Status in Primary Samples of HNSCC with the methylation of other eight genes.**

Patient and tumor Methylation

characteristics (***n*** = 165)

Present (***n*** = 107) Absent (***n*** = 58) ***P*-value**

*DCC methylation* †

Yes (78)

No (87)

*GALR1 methylation* †

Yes (81)

No (84)

*p16 methylation* †

Yes (73)

No (92)

*RASSF1A methylation* †

Yes (24)

No (141)

*CDH1 methylation* †

Yes (76)

No (89)

*CDH13 methylation* †

Yes (51)

No (114)

*MGMT methylation* †

Yes (58)

No (107)

*DAPK methylation* †

Yes (93)

No (72)

†Fisher’s exact probability test

56

51

68

39

48

59

13

94

59

48

41

66

43

64

76

31

22

36

13

45

25

33

11

47

17

41

10

48

15

43

17

41

0.102

< 0.001*

0.871

1

0.002*

0.005*

0.087

< 0.001*
