# Supplementary material for: Epigenetic silencing of SALL3 is an independent predictor of poor survival in head and neck cancer
Source: Clin Epigenetics. 2017 Jun 12;9:64. doi: 10.1186/s13148-017-0363-1 (PMC5469057; doi:10.1186/s13148-017-0363-1)
Supplement: Supplementary file 11 — SALL3 Gene Methylation levels in TCGA cohort of HNSCC (DOCX 21 kb). [file 13148_2017_363_MOESM11_ESM.docx]

**Table S4.** ***SALL3* Gene Methylation levels in TCGA cohort of HNSCC**

Patient and Variable (***n*** = 507)

*Age* †

70 and older (111)

Under 70 (396)

*Sex* †

Male (372)

Female (135)

*Smoking status* †

Smoker (379)

Nonsmoker (117)

　Unknown (11)

*Alcohol intake* †

Ever (338)

Never (158)

Unknown (11)

*Tumor size*†

T1-2 (179)

T3-4 (266)

　Unknown (62)

*Lympho-node status* †

N0 (173)

N+ (234)

Unknown (100)

*Stage*†

I, II, III (180)

IV (256)

Unknown (71)

*P16*†

Positive (36)

Negative (71)

Unknown (400)

† Student t test

*P<0.05.

*SALL3* methylation level ± SD

0.392 ± 0.190

0.338 ± 0.169

0.348 ± 0.173

0.354 ± 0.192

0.340 ± 0.186

0.383 ± 0.188

0.345 ± 0.186

0.361 ± 0.190

0.350 ± 0.179

0.359 ± 0.191

0.370 ± 0.188

0.339 ± 0.188

0.359 ± 0.186

0.352 ± 0.188

0.343 ± 0.187

0.362 ± 0.187

Data are expressed as means ± standard deviation.

***P*-value**

0.007*

0.718

0.030*

0.371

0.644

0.099

0.689

0.306
