# Supplementary material for: Epigenetic silencing of SALL3 is an independent predictor of poor survival in head and neck cancer
Source: Clin Epigenetics. 2017 Jun 12;9:64. doi: 10.1186/s13148-017-0363-1 (PMC5469057; doi:10.1186/s13148-017-0363-1)
Supplement: Supplementary file 12 — SALL3 mRNA levels in TCGA cohort of HNSCC (DOCX 23 kb). [file 13148_2017_363_MOESM12_ESM.docx]

**Table S5.** ***SALL3* mRNA levels in TCGA cohort of HNSCC**

Patient and Variable (***n*** = 507)

*Age* †

70 and older (111)

Under 70 (396)

*Sex* †

Male (372)

Female (135)

*Smoking status* †

Smoker (379)

Nonsmoker (117)

　Unknown (11)

*Alcohol intake* †

Ever (338)

Never (158)

Unknown (11)

*Tumor size*†

T1-2 (179)

T3-4 (266)

　Unknown (62)

*Lympho-node status* †

N0 (173)

N+ (234)

Unknown (100)

*Stage*†

I, II, III (180)

IV (256)

Unknown (71)

*P16 testing*†

Positive (36)

Negative (71)

Unknown (400)

† Student t tests

*P<0.05.

*SALL3* mRNA level ± SD

15.54 ± 83.71

20.86 ± 62.02

22.00 ± 86.58

13.39 ± 55.19

22.11 ± 89.05

12.64 ± 37.71

21.72 ± 88.82

16.70 ± 57.89

18.93 ± 63.32

18.99 ± 92.33

13.60 ± 54.23

21.16 ± 97.85

19.32 ± 65.60

17.93 ± 91.90

63.39 ± 117.38

5.23 ± 15.94

Data are expressed as means ± standard deviation.

***P*-value**

0.537

0.285

0.273

0.522

0.994

0.365

0.864

<0.001*
